# Evaluating the effectiveness of preventive training programs in reducing the incidence of knee injuries: a systematic review and meta-analysis

**DOI:** 10.3389/fpubh.2026.1746109

**Published:** 2026-03-23

**Authors:** Hao Zhou, Yu-Mei Xing, Dong-Liang Zhou, Long Cui, Jia Qian, Jun-Nan Li, Yi-Feng Bu

**Affiliations:** 1 School of Physical Education, Jiangsu Normal University, Xuzhou, China; 2 Library, Jiangsu Normal University, Xuzhou, China; 3 The Primary School Affiliated to Yunnan Normal University, Kunming, China

**Keywords:** athletes, knee injuries, prevention and control, prophylaxis, random control trails

## Abstract

**Introduction:**

Knee injuries are highly prevalent among athletes. Following a knee injury, athletes may experience minor disruptions to their competition and training schedules, or in severe cases, the injury may even jeopardize their athletic careers. Incorporating targeted knee injury prevention training into routine training is thus critical for mitigating knee injury risk.

**Aim:**

This meta-analysis aimed to investigated whether exercise-based preventive training influences the incidence of knee injuries in athletes. Exploratory and prespecified subgroup analyses were concurrently performed to identify potential factors influencing the effectiveness of preventive training programs.

**Method:**

This study adopted a systematic review and meta-analysis design. A systematic literature search was performed across four electronic databases: PubMed, Embase, Web of Science, and Cochrane Library. The main search terms included “Athletes,” “Knee Injuries,” “Prevention and Control,” and “Random Allocation.”

**Results:**

A total of 10 studies, involving 15,296 participants, were included in this meta - analysis. The groups that received preventive training showed a significantly lower incidence of knee injuries (overall; RR = 0.68, 95% CI: 0.51–0.92, *p* = 0.011) and anterior cruciate ligament injuries (RR = 0.43, 95% CI: 0.25–0.74, *p* = 0.002). Subgroup analysis also revealed several group differences; however, these findings should be interpreted with caution due to the small sample size. The overall heterogeneity of the results was high, and the sources of heterogeneity could not be identified.

**Conclusion:**

Exercise-based injury-prevention training (such as strength, balance, and plyometric training) can reduce the incidence of knee injuries.

**Systematic review registration number:**

PROSPERO (registration no. CRD42024543884).

## Introduction

As competitive sports continue to evolve, athletes' overall performance levels are steadily improving, and the intensity of competition is growing increasingly fierce. Against this backdrop, some training teams have begun to adjust training load and intensity to enhance athletes' competitive edge. While this approach does boost athletes' performance to a certain extent, it also elevates their risk of sports-related injuries (1). A 2012 study of load and soft tissue injuries in athletes revealed that elite rugby players who performed more hyper speed-related exercises per training session were 2.7 times more likely to suffer an injury than those who did not (2). Similarly, the Australian Football Club Federation launched research on the relationship between sports load and injury rate in 2011. The researchers reported that 40% of the injuries were associated with a significant increase (>10%) in the training load during the previous week (3). Although high-intensity training is associated with elevated injury rate, an excessive focus on the adverse effects of such training may also compromise the positive physiological adaptations induced by the training process (1). Therefore, greater emphasis should be placed on the research and implementation of preventive training protocols.

Establishing a definitive link between training load and sports injuries is a fundamental prerequisite for designing scientifically sound training protocols; accurate localization of injury sites is critical to optimizing the target specificity and effectiveness of intervention approaches. Knee joint injuries stand out as a particularly prevalent issue among all athletic injuries, which calls for thorough and systematic analysis.

The knee joint represents a primary focus in the management of sports-related injuries. Such injuries are not only among the most prevalent conditions in competitive sports but also exert a profound impact on an athlete's entire professional career (4, 5). For instance, anterior cruciate ligament (ACL) injuries are often referred to as career breakers for athletes. In the United States alone, more than 250,000 ACL related injuries are reported annually, with over $1 billion spent each year on the rehabilitation and surgical reconstruction of the affected knees (6, 7). Among them, the ACL injury rate is 3.5% in female athletes and 2% in male athletes (8). Most of them lose athletic scholarships or contracts because they take too long to recover (8). Most ACL injuries are non-contact injuries. When the knee joint is subjected to torsion or shear force, the ACL bears a large load and is prone to injury (5, 9). Athletes are required to execute these movements frequently and efficiently in training and competition, which undoubtedly elevates their risk of injury. Additionally, athletes who frequently rely on explosive jumping are susceptible to knee joint degenerative disorders, quadriceps muscle avulsions, and other related injuries, owing to the sustained high loads imposed on the patellar tendon over prolonged periods (10, 11). Meniscus tears are more common among athletes. Approximately one-third of patients with meniscal tears that require meniscal surgery are the result of exercise (12).

However, a series of injury problems caused by high-intensity training are not inevitable. A well-designed set of training guidelines can not only maximize athletes' competitive performance but also mitigate the adverse effects associated with training (1). In addition to the rational regulation of training load and rest intervals, the integration of injury prevention training programs has also been proven clinically effective. For instance, Fédération Internationale de Football Association (FIFA) 11+ is a systematic warm-up training that has been shown to have a good preventive effect on the injury of football players (13, 14). An amateur men's soccer team from Italy implemented a 9-week FIFA 11+ training intervention, and subsequent assessments demonstrated significant improvements in the athletes' neuromuscular control and competitive performance (15). This training technique not only improves the muscle strength of the athlete but also prepares the athlete for competition, thus greatly reducing the possibility of injury (14). Neuromuscular training represents a widely adopted injury prevention program. Participants are instructed to prioritize movement quality while strengthening the relevant muscle groups, so as to mitigate injury risk. For instance, a 2015 Danish ACL injury prevention program targeting female athletes incorporated this training modality. The findings indicated that such preventive interventions can optimize athletes' muscle activation patterns during specific movements, thereby reducing the incidence of ACL injuries (16). In addition, approximately 16% of muscle injuries during sports are secondary injuries (17). If such athletes add some centrifugal training or core stabilization training for weak muscle groups after the initial injury, the risk of secondary injury can be reduced (14, 18–20). However, the findings derived from randomized controlled trials (RCTs) are confined to specific intervention strategies and narrowly defined participant cohorts. To date, meta-analyses focusing on knee injury prevention in athletic populations have either been confined to a single sport or limited to the investigation of only one type of knee injury (19, 21). We therefore aimed to expand this scope by conducting a meta-analysis without restricting the types of athletes or specific knee injuries involved.

Therefore, the aim of this meta-analysis was to comprehensively examine the effects of preventive training on knee joint injuries. At the same time, injury prevention programs yield heterogeneous effects depending on athletes' characteristics, gender, and injury time points. Therefore, we also conducted an exploratory subgroup analysis to tentatively investigate the potential differences across these subgroups.

## Method

### Registration

This study is a systematic review and meta-analysis. The protocol of this systematic review and meta-analysis has been registered on the PROSPERO website (registration no. CRD42024543884) and follows the guidelines of Systematic Reviews and Meta-Analyses (22).

### Data sources and search strategy

We systematically searched four databases: PubMed database, Web of Science Core Collection database, Embase database, and Cochrane Library. The search covered the period from 1 January 2000 to 1 May 2025 (Final search date: 1 May 2025). The search strategy combined Mesh terms and free-text keywords to identify literature related to knee-injury prevention in athletes. The selected MeSH terms include: “Athletes,” “Knee injuries,” “Prevention and control,” and “Random Allocation.” The full search strategy is provided in Supplementary File 1. Two researchers independently searched for potentially relevant English articles. If there was any disagreement, a third person participated in the discussion.

### Eligibility criteria

The specific selection criteria were as follows: (1) the subjects were athletes; (2) the interventions of the experimental groups were related to injury prevention programs, such as sports intervention (neuromuscular training, FIFA 11+ training, core training, etc.), safety education and error pattern correction; (3) the control groups kept the original training that was not exposed to any special intervention; (4) the included studies were RCTs published between 2000 and 2025; (5) the included articles must undergo peer review; (6) article must be published in English; (7) reported the number of knee and lower extremity injuries in results. The exclusion criteria were as follows: (1) non-athletes among the subjects; (2) < 20 athletes in each group (if the sample size was too small; we believe that the quality of the results was low, and there may be particularities and chance involved) (23); (3) prevention training was not implemented in the intervention group, or was additionally administered in the control group; (4) outcomes related to knee or lower limb injury for which the outcome measures were not reported; (5) case reports, animal experiments, review articles, and unpublished articles.

### Study selection

Two independent researchers first selected suitable articles after reviewing the article titles and abstracts. After that, the full texts were evaluated against the selection criteria to determine whether potentially relevant articles were eligible for final inclusion. In case of discrepancies, a third researcher will join the discussion to resolve the issue. Studies were included if they met the predefined eligibility criteria. The articles in this study included male and female athletes and did not control for age or sports. For articles where there was no way to obtain detailed information from the original text, we attempted to address this problem by contacting the authors or finding additional documents. If both approaches failed, then this article was excluded.

### Data extraction

Two researchers independently extracted data from the selected RCTs with disagreements resolved through discussion. First, baseline data, which included author, country, athlete type, height and weight, sample size, intervention style, intervention length, and reported outcomes, was extracted. Second, to better reflect the intervention methods and results reported in the original article, we decided to make a separate table showing the intervention details, exercise exposure time and reported injuries of each study. All injuries included knee injuries, lower-limb injuries and whole-body injuries because of differences in research reports. In the event of discrepancies between two researchers, a third researcher will be consulted to resolve the issue through discussion.

### Outcomes

The primary outcomes of this study included knee injuries (overall) and ACL injuries, quantified by the number of occurrences. Knee injuries (overall) reported in the original studies were acute or overuse injuries sustained during competition and training that required medical intervention. Articles reporting relevant outcomes documented the incidence of such injuries during the intervention period. We extracted these incidence data and compared them with those of the control group. It should be noted that the definition of ACL injuries was consistent across all included studies, and the gold standard for diagnosis is magnetic resonance imaging. For secondary outcomes, we extracted the total number of injuries across all categories. Although each article had a distinct definition of injury and not every article reported overall injury rate, all included articles provided data on major injuries. Finally, the overall injury cases in each study may have comprised knee injuries, lower-extremity injuries, and whole—body injuries. To account for variations in injury definitions, we will report the specific injury types included in each study in a table. This heterogeneity will also be explicitly stated as a limitation of the present study in the Limitations section. Furthermore, three subgroup analyses were performed to identify injury related differences stratified by sex, type of athlete, and occasion of injury onset. The risk ratio was used for analysis and comparison. However, due to the reporting format of the original articles, we instead analyzed the reported number of injuries.

### Risk of bias (RoB)

Two independent reviewers assessed the RoB of the included RCTs using the Cochrane Risk-of-Bias 2.0 tool, BMJ (24). They evaluated each study across five key domains: the randomization process, deviations from intended interventions, missing outcome data, measurement of outcomes, and the selection and reporting of results, including any protocol deviations. For every domain, reviewers assigned “low,” “some concerns,” or “high” risk according to the predefined signaling questions and algorithms in RoB 2.0. Overall judgments were derived algorithmically from the domain-level ratings. If a study showed a high risk in any single domain, it was classified as having an overall high risk of bias. Conversely, studies with low risk across all domains were categorized as having an overall low risk. Discrepancies at both domain and overall levels were resolved by consensus.

### Data analysis

Following data extraction, heterogeneity among studies was evaluated using Cochran's *Q*-test and Higgins' *I*^2^ statistic. A *p*-value less than 0.10 or an *I*^2^ greater than 50% was interpreted as evidence of substantial heterogeneity, prompting the use of a random-effects model; otherwise, a fixed-effects model was applied. Meta-analyses were performed using Stata 15.0 StataCorp LLC (College Station, Texas) to assess the effects of injury prevention programs on injury rate, with rate ratios and 95% confidence intervals (CIs) reported for binary outcomes. Given that some of the included randomized controlled trials were cluster-randomized, we performed a sensitivity analysis across all outcomes to examine the potential impact of this design on the study findings. Meanwhile, We explored potential sources of heterogeneity through additional sensitivity analyses and subgroup analyses. These subgroup comparisons (i.e. occasion of injuries, type of athlete, and gender) were exploratory and *post hoc*. Statistical significance was determined at *p* < 0.05. Publication bias was assessed both visually with funnel plots and statistically using Egger's and Begg's tests. Additionally, the trim-and-fill method was applied to evaluate and adjust for potential publication bias in the meta-analysis findings.

## Results

### Selection results

**Figure 1:**
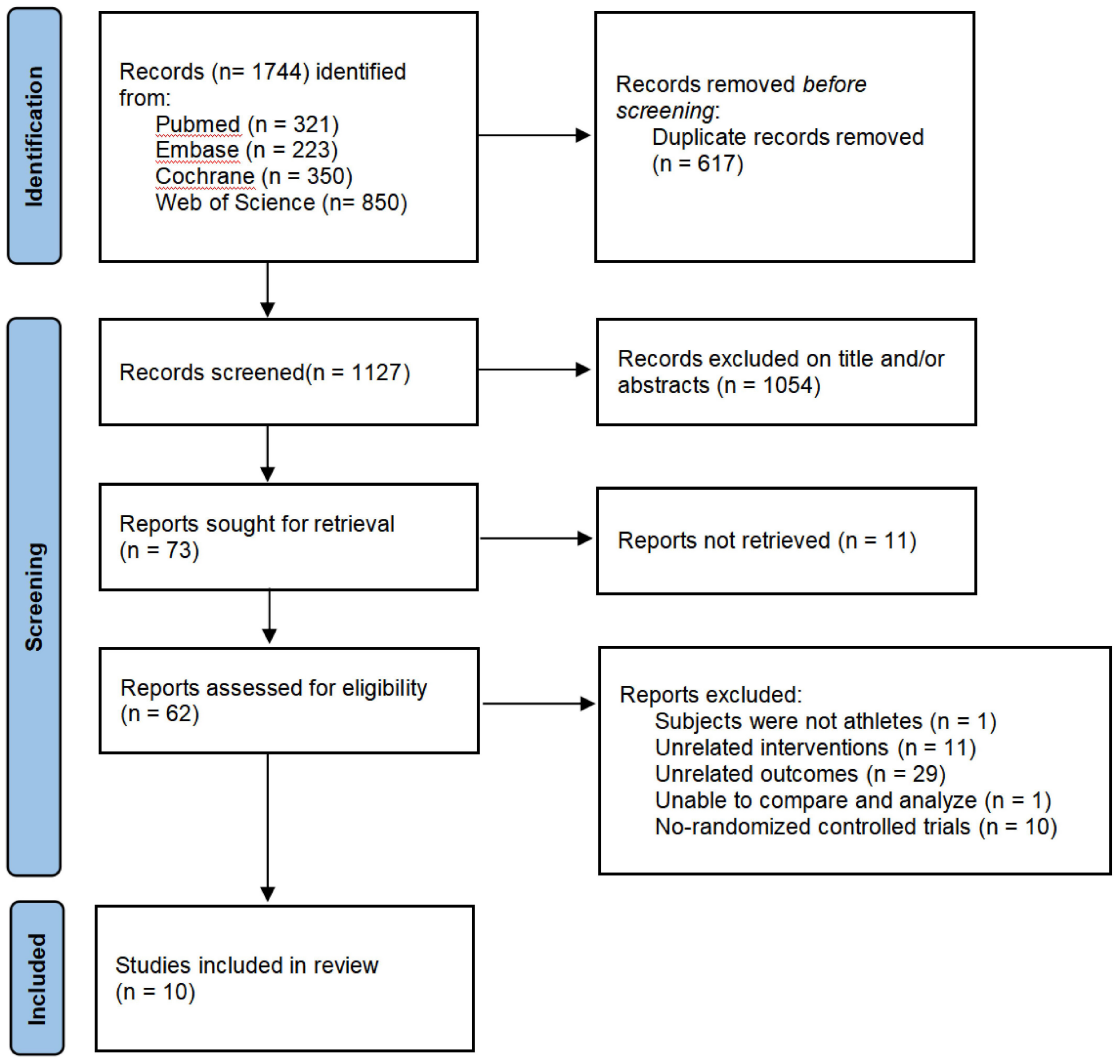
Flow chart of the selection process.

The study selection process is illustrated in Figure 1. Of the 1,744 records initially identified, 617 were excluded due to duplicate records, irrelevant titles, and/or ineligible abstracts. Full-text assessment was conducted for the remaining 73 articles, of which 52 were subsequently excluded, primarily because they did not employ a RCT design or reported outcome measures unrelated to injury prevention. Ultimately, 10 studies examining injury-prevention programs in athletic populations were included in the final analysis (25–34). It should be noted that all subgroup analyses were exploratory and prespecified.

### Study characteristics

Table 1 presents the basic characteristics.

**Table 1:** The characteristics included in the study.

**Time**	**Country**	**Type of athlete**	**Age, mean**	**Sample size (number of male)**	**Intervention (G1)**	**Intervention length**	**Follow-up time**	**Outcomes**	**Overall injury definition or inclusion**	**Randomization type**
Gilchrist et al. (26)	USA	Soccer	G1 = 19.88 G2 = 19.88	G1 = 583 (0) G2 = 852 (0)	Prevent injury and Enhance Performance	12 weeks	NR	1. Exposure time for training and competition 2. Knee injuries and ACL injuries 3. Injury rate per 1,000 AE	Including all injuries of knee joint	Cluster randomized
Kiani et al. (27)	Sweden	Soccer	G1 = 14.7 G2 = 15	G1 = 777 (0) G2 = 729 (0)	The Harmoknee preventive training program	9 months	6 months	1. Exposure time for training and competition 2. Knee injuries and injury diagnoses 3. Injury rate per 1,000 h 4. Injuries in games or practices	Including all injuries of the knee joint	Cluster randomized
LaBella et al. (29)	USA	Soccer, basketball	G1 = 16.19 G2 = 16.22	G1 = 737 (0) G2 = 755 (0)	Coach-led neuromuscular warm-up	A season	NR	1. Knee injuries and ACL injuries 2. Type of injuries (gradual, acute)	Including all lower extremity injuries	Cluster randomized
Longo et al. (30)	UK	Basketball	G1 1 3.5 G2 = 15.2	G1 = 80 (80) G2 = 41 (41)	FIFA 11 + program	A season	9 months	1. Exposure time for training and competition 2. All injuries and knee injuries 3. Acute injuries and overuse injuries 4. Injuries of varying severity	Including all lower extremity injuries	Cluster randomized
Krist et al. (28)	Netherlands	Soccer	G1 = 24.4 G2 = 25.1	G1 = 223 (223) G2 = 233 (233)	The injury prevention program during the warm-up	33 weeks	NR	1. All injuries 2. All injured players 3. Injury related medical expenses	Including all injuries that necessitate medical intervention or lead to the inability to resume participation in relevant activities	Cluster randomized
Waldén et al. (34)	Sweden	Soccer	G1 = 14 G2 = 14.1	G1 = 2,479 (0) G2 = 2,085 (0)	Neuromuscular warm-up program	7 weeks	NR	1. ACL injuries 2. Knee injuries 3. Sever knee injuries and acute knee injuries	Including all injuries of the knee joint	Cluster randomized
Silvers-Granelli et al. (31)	USA	Soccer	G1 = 20.68 G2 = 20.4	G1 = 675 (675) G2 = 850 (850)	FIFA 11+ injury prevention program	4 months	NR	1. Injury rate per 1,000 AE 2. All injuries 3. Injuries in games or practices 4. Different ares of the injuries	Including injuries involving the following anatomical locations: ankle, knee, head, hamstring, foot, groin, hip, quadriceps, leg, shoulder, spine, hand, torso, elbow, wrist, neck, chest, arm, forearm.	Cluster randomized
Foss et al. (25)	USA	Soccer, basketball, volleyball	G1 = 14 G2 = 14	G1 = 259 (0) G2 = 215 (0)	Core intervention	A season	NR	1. All injuries 2. Knee and Patellar Injuries 3. Injuries to athletes of different grades	An injury was defined as any of the following circumstances: 1. any injury causing cessation of participation in the current session; 2. any injury that caused cessation of participation on the day after the day of onset; 3. any fracture; 4. any dental injury; 5. any mild brain injury regardless of time missed from participation.	Individual randomized
Slauterbeck et al. (32)	USA	Soccer, basketball, football, hockey	NR	G1 = 1,825 (NR) G2 = 1786 (NR)	The FIFA 11+	A season	NR	1. All injures 2. Knee injures 3. Injury rate per 1,000 AE 4. Type of injuries (fracture, bruise etc.)	Including all injuries of the knee joint	Cluster randomized
Stojanović et al. (33)	Switzerland	Basketball	G1 = 21.6 ± 2.5 G2 = 21.6 ± 2.6	G1 = 57 (42) G2 = 55 (43)	Multicomponent neuromuscular warm-up program	7 months	NR	1. All injuries 2. Knee injuries and ACL injuries 3. Injury rate per 1,000 AE	Including injuries involving the following anatomical locations: achilles tendinitis, ankle sprain, ACL tear, knee sprain, overall knee, quadriceps contusion, hamstring strain.	Cluster randomized

NR, no report; AE, athlete exposures; ACL, anterior cruciate ligament; FIFA, Fédération Internationale de Football Association; G1, injury-prevention group; G2, control group.

The articles we considered covered 15 years (published from 2008 to 2023), and the number of participants in the studies varied from 112 to 4,564. Out of these, five studies were done in the United States (25, 26, 29, 31, 32), and five were conducted in Europe (27, 28, 30, 33, 34). Most studies focused on athletes from just one sport (26–28, 30, 31, 33, 34), with football and basketball being the most common, while three studies included athletes from more than one sport (25, 29, 32). Five articles examined female athletes (25–27, 29, 34), three looked at male athletes (28, 30, 31), and only one did not differentiate between genders (one did not specify gender) (33). The treatment period ranged from 7 to 36 weeks, with most treatments lasting for one season. The prevention programs consist of warm-up exercises, and most of these exercises involve neuromuscular training, as detailed in Table 2.

**Table 2:** The characteristics of prevention programs.

**Author**	**Experimental group intervention details**	**Exposure hours**	**All injuries**	**Knee injuries**	**ACL injuries**
Gilchrist et al. (26)	1. Warm-up (50 yards each); 2. Stretching (30 s × 2 reps each); 3. Strengthening; 4. Plyometrics (20 reps each); 5. Agilities.	G1 = 35,220^a^ G2 = 52,919^a^	G1 = 40 G2 = 58	G1 = 40 G2 = 58	G1 = 7 G2 = 18
Kiani et al. (27)	1. Warm-up (≥10 min); 2. Muscle activation, contracting the muscle for 4 s each movement; 3. Balance, each action maintaining approximately 30 s; 4. Strength (three actions); 5. Core stability (three actions).	G1 = 66,981 G2 = 66,505	G1 = 3 G2 = 13	G1 = 3 G2 = 13	G1 = 0 G2 = 3
LaBella et al. (29)	20-min neuromuscular warm-up combining progressive strengthening, plyometric, balance, and agility exercises.	G1 = 28,023^a^ G2 = 22,925^a^	G1 = 50 G2 = 96	NR	G1 = 2 G2 = 6
Longo et al. (30)	1. Running exercises, 8 min (along the major diameter of the basketball court, about 28 meters); 2. Strength, plyometrics, balance, 15 min; 3. Running exercises, 1 min and 40 s (along the major diameter of the basketball field, about 28 m).	G1 = 23,640 G2 = 12,648	G1 = 14 G2 = 17	G1 = 5 G2 = 2	NR
Krist et al. (28)	11 injury prevention program contains 10 exercises (the bench, sideways bench, hamstrings, cross country skiing, chest-passing in single-leg stance, forward bend in single-leg stance, figures-of-eight in single-leg stance, jumps over a line, zigzag shuffle, bounding).	NR	G1 = 207 G2 = 220	NR	NR
Waldén et al. (34)	1. One legged knee squat; 2. Pelvic lift; 3. Two-legged knee squat; 4. The bench; 5. The lunge; 6. Jump/landing; (Each movement consists of four levels and a pair-exercise).	G1 = 149,214 G2 = 129,084	G1 = 49 G2 = 47	G1 = 49 G2 = 47	G1 = 7 G2 = 14
Silvers-Granelli et al. (31)	1. Running exercises, encompass cutting, change of direction, decelerating, and proper landing techniques (8 min); 2. Strength, plyometric, and balance exercises (2 min); 3. Running exercises, to conclude the warm-up and prepare the athlete for athletic participation (2 min); Each specific exercise has three levels.	G1 = 35,226^a^ G2 = 44,212^a^	G1 = 285 G2 = 665	G1 = 34 G2 = 102	NR
Foss et al. (25)	1. Exercises completed in the preseason, containing 13 actions; 2. Exercises completed in-season, containing seven actions.	G1 = 22,906^a^ G2 = 19,875^a^	G1 = 107 G2 = 134	G1 = 60 G2 = 72	NR
Slauterbeck et al. (32)	FIFA 11+ program including exercises to increase strength, improve coordination, and enhance the running strategy.	G1 = 116,079^a^ G2 = 113,420^a^	G1 = 196 G2 = 172	G1 = 35 G2 = 41	NR
Stojanović et al. (33)	1. Running combined with active stretching (containing six actions); 2. Plyometrics, balance, and strength (containing 10 action, each with three levels); 3. Agility (containing two actions).	G1 = 11,268.5 G2 = 10,737.5	G1 = 6 G2 = 20	G1 = 2 G2 = 6	G1 = 1 G2 = 3

NR, no report; ACL, anterior cruciate ligament; G1, injury-prevention group; G2, control group.^a^represents: Calculated from athletic exposures (1 athletic exposure = 2 exposure hours).

Meanwhile, given the discrepancies in the reporting of raw research data, we specifically designed a table to elaborate on the composition of overall injuries reported in each study. In addition, we also clarified the details of randomization for group allocation.

### Risk of bias

**Figure 2:**
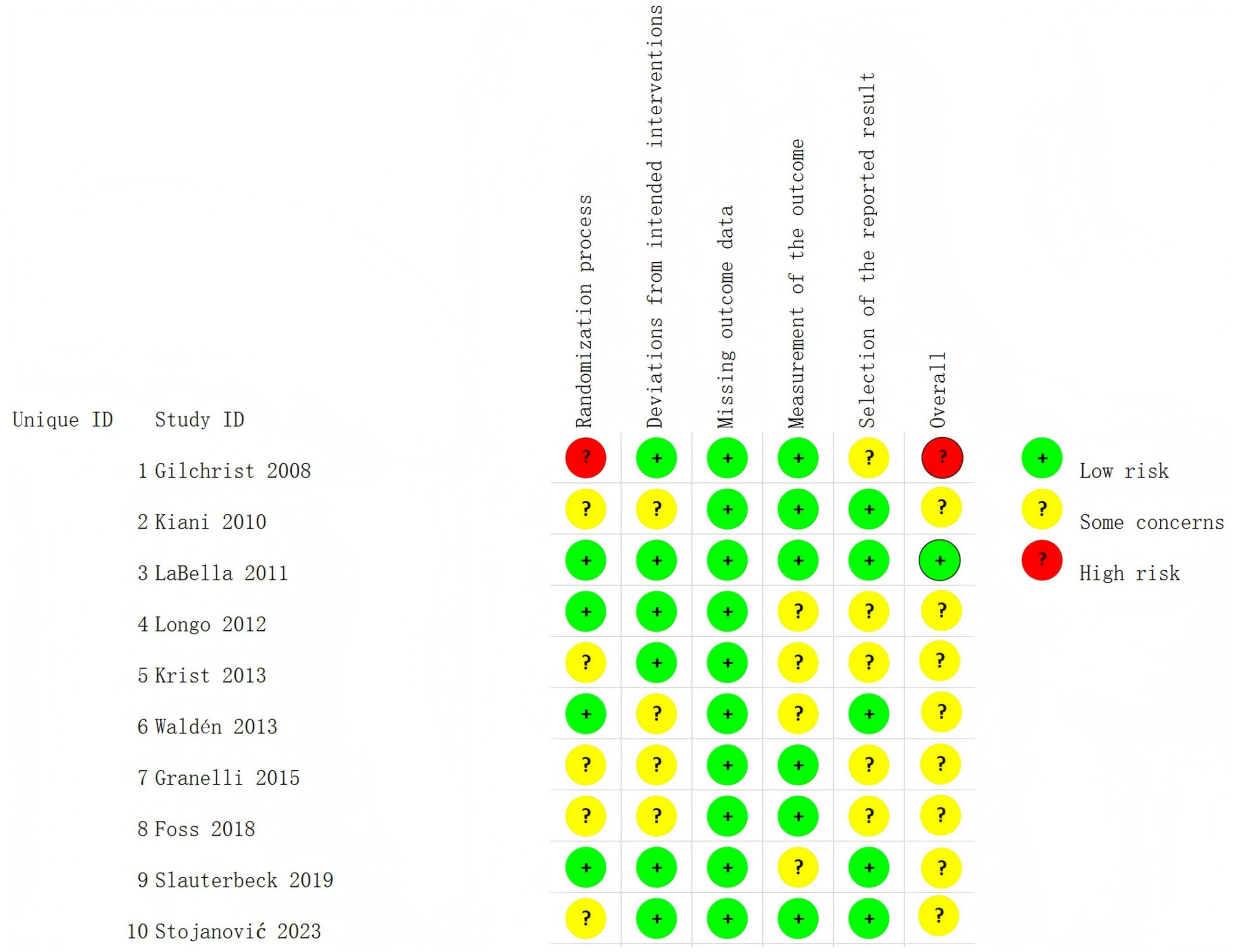
Risk of bias result.

The RoB result was shown in Figure 2. All the studies explained how they created random sequences. Most studies kept the subjects unaware of their group, but only one study specifically mentioned that the researchers were also kept unaware (34). None of the studies mentioned if the person who measured the results knew which group the subjects were in. The sole study with high risk of bias was attributed to unclear randomization procedures. After integrating all domain-level judgments, we rated eight studies as “some concerns” (25, 27, 28, 30–34), one as low RoB (29), and only one as high RoB (26) (Figure 2). The two evaluators agreed on these results. These sources of bias may exert a certain impact on the findings of the present study, thus reducing the overall quality of evidence.

### Effects of interventions on knee injuries (overall) and ACL injuries

**Figure 3:**
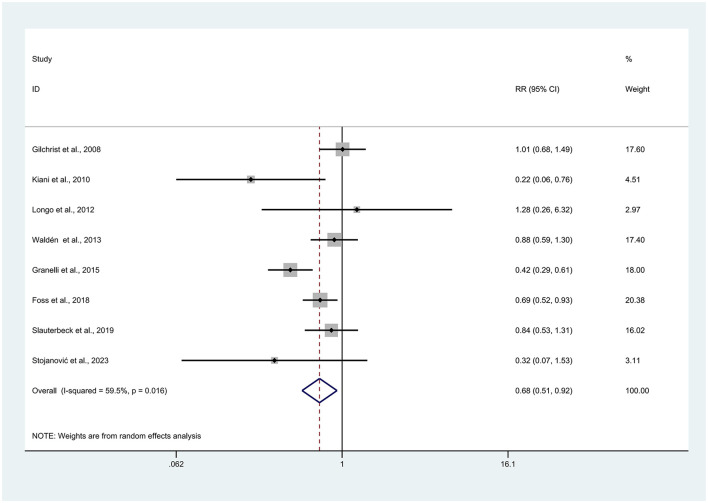
Forest plot of knee injuries (overall) risk. Risk ratios (RRs) with 95% confidence intervals (CIs) are presented using a random-effects model; heterogeneity is expressed as *I*^2^.

Eight studies mentioned knee injuries (7, 25–27, 30, 32–34). Owing to the high heterogeneity (*I*^2^ = 59.5%), a random effects model was used for analysis. Compared with the control group, the experimental group presented a significantly lower number of knee injuries (RR = 0.68, 95% CI: 0.51–0.92, *p* = 0.011; Figure 3 about here).

**Figure 4:**
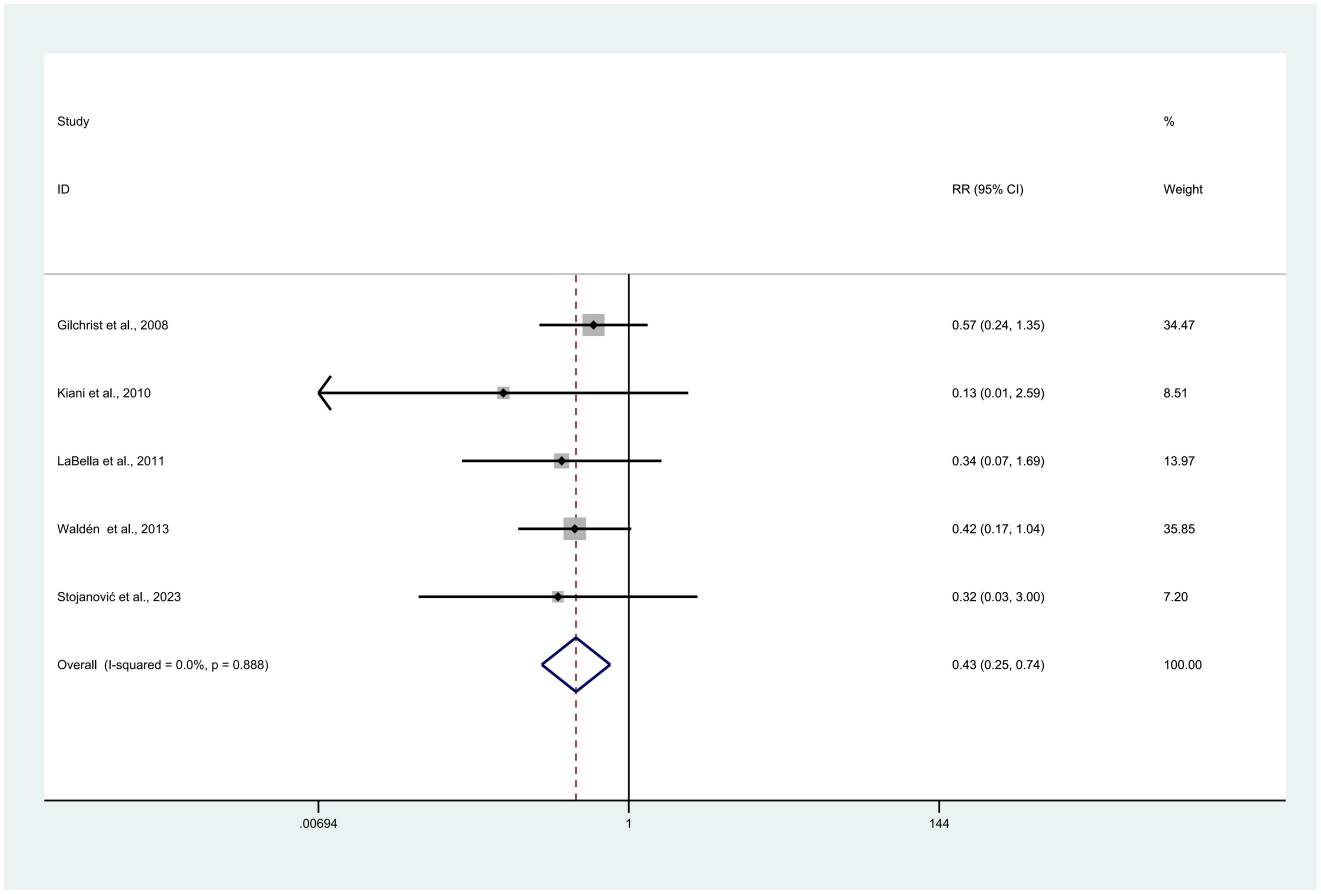
Forest plot of anterior cruciate ligament injuries risk. Risk ratios (RRs) with 95% confidence intervals (CIs) are presented using a Fixed effects model; heterogeneity is expressed as *I*^2^.

Of the included articles, five specifically reported data on ACL injuries (26, 27, 29, 33, 34). After the heterogeneity test, low heterogeneity was found (*I*^2^ = 0.0%), so the fixed effects model was used for analysis. Injury prevention programs had a statistically significant effect on reducing the number of ACL injury events in athletes (RR = 0.43, 95% CI: 0.25–0.74, *p* = 0.002; Figure 4).

### Relationship between occasion of injuries and injuries

**Figure 5:**
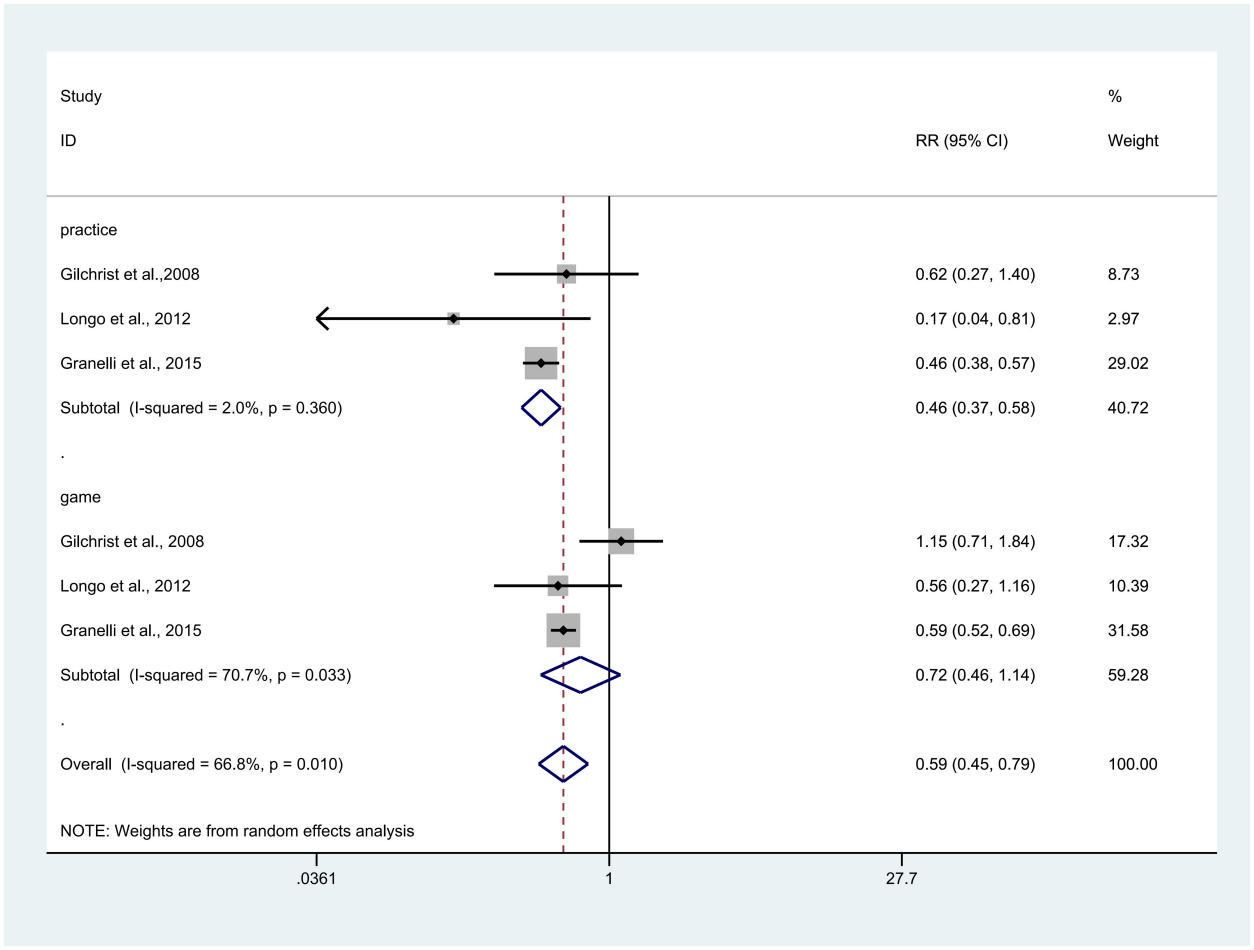
Forest plot of injury risk stratified by occasion of injuries (practice vs. game). Risk ratios (RRs) with 95% confidence intervals (CIs) are presented using a random-effects model; heterogeneity is expressed as *I*^2^.

Athletic injuries can occur at any stage of training or competition, and the injuries documented in the present study were sustained during both practice sessions and official matches. A total of six related results were reported in three articles (26, 30, 31), and due to high heterogeneity (*I*^2^ = 66.8%), a random effects model was selected for analysis. The results revealed that the experimental group had a significant reduction in the occurrence of injury during both practice sessions and official matches (RR = 0.59, 95% CI: 0.45–0.79, *p* < 0.001). After subgroup analysis, we found that the degree of injury occurrence in the experimental group was significantly lower than that in the control group (RR = 0.46, 95% CI: 0.37–0.58, *p* < 0.001) during the practice sessions. However, the difference was not significant (RR = 0.72, 95% CI: 0.46–1.14, *p* = 0.195) during the official matches (Figure 5). Note: This subgroup analysis is exploratory.

### Relationship between type of athlete and injuries

**Figure 6:**
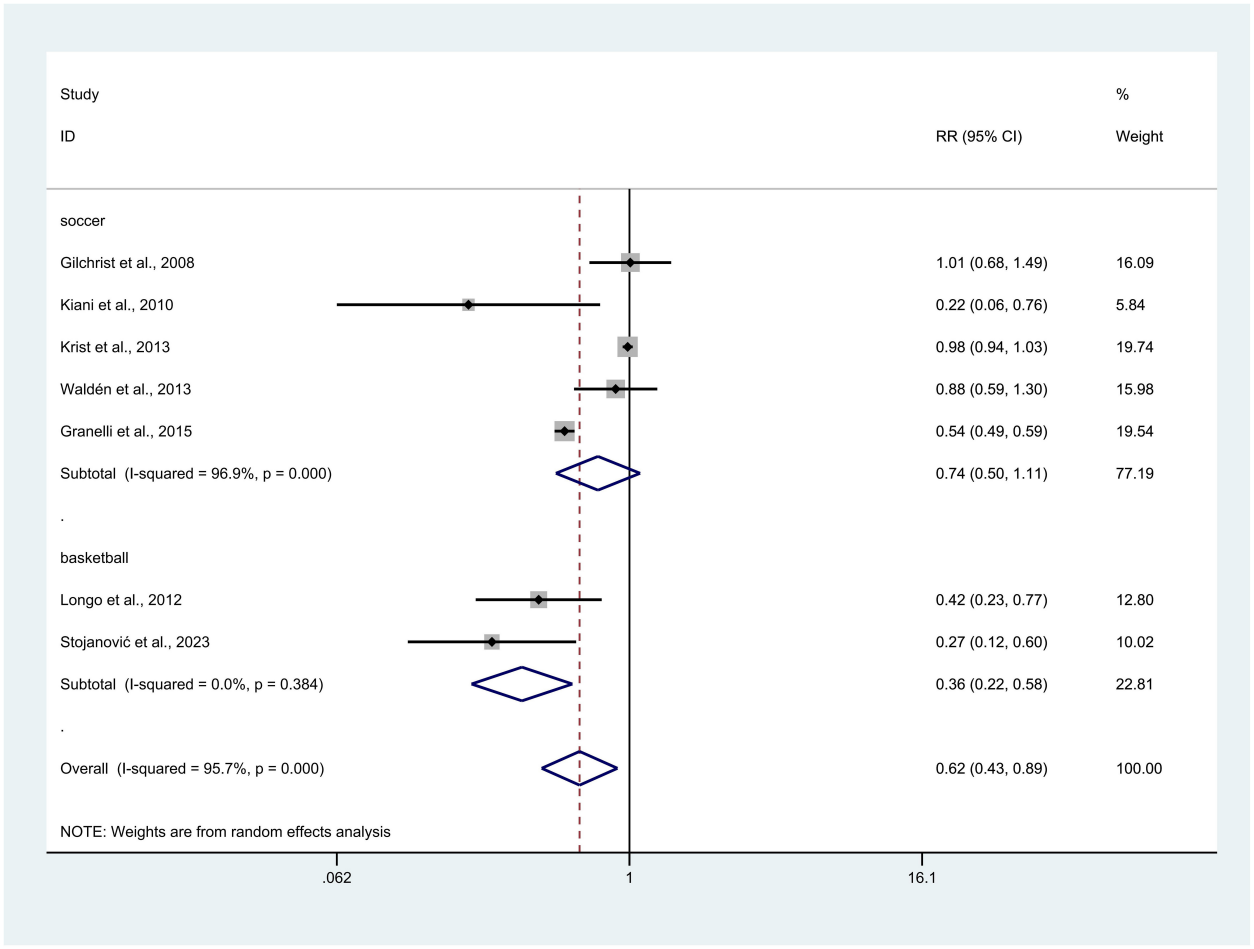
Forest plot of injury risk stratified by type of athlete. Risk ratios (RRs) with 95% confidence intervals (CIs) are presented using a random-effects model; heterogeneity is expressed as *I*^2^.

A total of two different sports types were extracted, with five articles on football players (26–28, 31, 34), and two articles on basketball players (30, 33). The random effects model was selected because of high heterogeneity (*I*^2^ = 95.7%). After subgroup analysis of the results, we found that prevention training was significant for injury reduction among basketball players (RR = 0.36, 95% CI: 0.22–0.58, *p* < 0.001), whereas it was not significant for football players, with a better trend (RR = 0.74, 95% CI: 0.50–1.11, *p* = 0.143). Overall, the data reporting this result showed that prevention programs reduced injuries occurrence in athletes (RR = 0.62, 95% CI: 0.43–0.89, *p* = 0.01; Figure 6). Note: This subgroup analysis is exploratory.

### Relationship between sex and injuries

**Figure 7:**
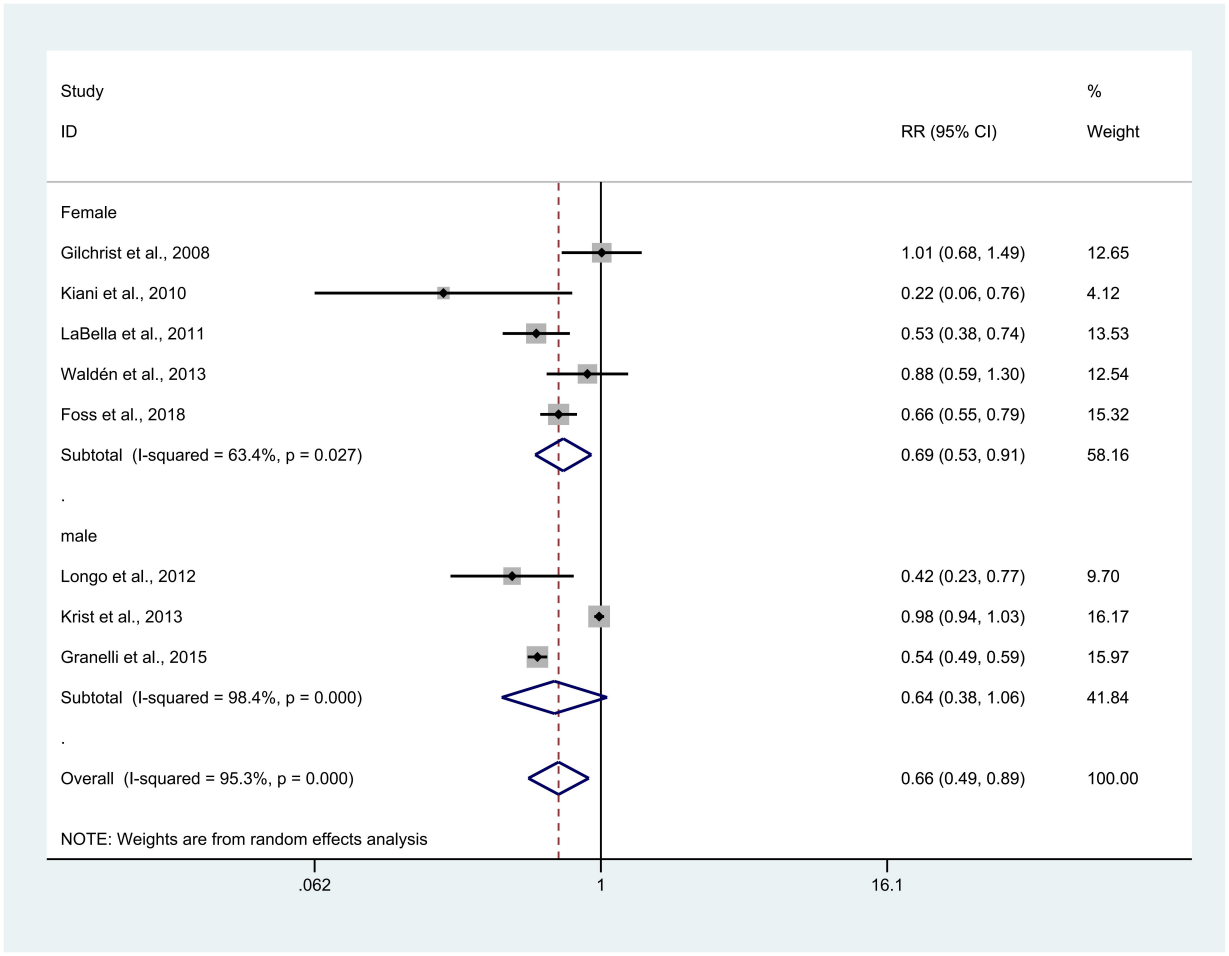
Forest plot of injury risk stratified by sex. Risk ratios (RRs) with 95% confidence intervals (CIs) are presented using a random-effects model; heterogeneity is expressed as *I*^2^.

A total of eight articles reported sex-related results, and due to high heterogeneity (*I*^2^ = 95.7%), a random effects model was used for analysis. Five articles on female athletes revealed that the experimental group had a significantly lower incidence of sports injuries compared to the control group (RR = 0.69, 95% CI: 0.53–0.61, *p* = 0.009) (25–27, 29, 34). The difference was not significant across the three articles about male sports (RR = 0.64, 95% CI: 0.38–1.06, *p* = 0.084) (28, 30, 31) (Figure 7). Note: This subgroup analysis is exploratory.

### Sensitivity analysis

Each result was subjected to sensitivity analysis. By individually removing one study at a time, sensitivity analysis revealed no statistically notable changes in the meta-analysis results. This indicates that no experimental data need to be excluded.

### Publication bias

First, a funnel plot was used to intuitively reflect the publication bias of each result. Formal tests did not detect asymmetry, but the small number of included studies limits the power of these analyses; the presence of publication bias cannot be excluded [knee injuries (overall): Begg's test = 1, Egger's test = 0.88; ACL injuries: Begg's test = 0.086, Egger's test = 0.101; occasion of injuries: Begg's test = 0.707, Egger's test = 0.705; type of athlete: Begg's test = 0.548, Egger's test = 0.373; sex: Begg's test = 0.711, Egger's test = 0.246]. Nevertheless, the limited number of included studies has to a certain extent constrained the interpretive power of these testing methods.

## Discussion

### Summary of principal findings

Knee injuries are a frequent and significant issue for athletes, but specific injury prevention programs can greatly lower the risk of these injuries (35). This study systematically reviewed existing literature on athletic injury prevention training programs. Our findings indicated that athletes who engaged in targeted preventive training programs exhibited significantly lower incidence of ACL injuries and overall knee injuries than controls. Although the protective benefits varies across athlete groups and competitive settings, these results highlight the clinical relevance and practical value of structured injury prevention training programs.

### Interpretation of prevention programs on ACL injuries

Preventive training programs can effectively reduce the incidence of ACL injuries in athletes. These findings are consistent with the conclusions of previous meta-analyses focusing on exercise-based ACL injury prevention programs (36–39). The main function of the ACL is to stop the shinbone from moving too far forward (40). During high-risk movements such as single-leg landing, rapid speed changes, or sudden deceleration, the ACL sustains elevated mechanical loads that can precipitate acute rupture and elevate the overall risk of musculoskeletal damage (41–43). For most athletes, these actions are hard to avoid. All injury prevention programs in the present study were multicomponent interventions, incorporating both strength training and progressive overload exercises. Strength training is critical for athletes to sustain high-level athletic performance and achieve progressive adaptations (44). Targeted training of the musculature surrounding the knee joint can not only attenuate mechanical loading on the ACL during high-risk movements but also facilitate the execution of more precise motor patterns in athletes (45). Previous studies showed that the maximum outward twisting force of the knee joint decreases after preventive training (46), indicating a reduced risk of ACL injuries (47, 48). Furthermore, progressive training can help athletes better adapt to a wide range of scenarios. Muscle hypertrophy and the ability to withstand external shocks are largely determined by the intensity of the normal load received (49). Similarly, the process of muscle growth also arises from the adaptation of different intensity loads over and over (49, 50). The present study showed that systematic preventive training programs has enabled athletes to develop the capacity to resist ACL injuries. Notably, poor adherence to the training will diminish the preventive benefit (51, 52).

### Interpretation of prevention programs on knee injuries (overall)

In 2015, 10%−25% of injuries reported in young athletic populations were diagnosed as knee injuries (53). Preventive training can lower the rate of knee injuries in sports groups, which matches the findings of earlier studies (19, 21, 36, 38). However, few previous articles have explained these results. We analyzed different components of the prevention program: strength training, engagement of the knee joint, and consideration of the prevention program as a warm-up. Strength training strengthens the surrounding muscles, which may be a key point (49). Physiologically, muscle growth increases the size of muscle cross-sections, giving athletes more strength to perform movements and avoid injuries from weak muscles (41, 49, 54). Moreover, knee joint is a structurally and biomechanically complex synovial joint, involving in the majority of lower-extremity motor patterns (5, 55). Enhancing neuromuscular control optimizes knee stability and function, providing a robust defense against injury (56). Besides, preventive training programs can be viewed as warm-up exercises. Literature has shown that warm-ups reduce musculoskeletal injuries (57). During warm-up, muscle activity improves, and muscle stiffness decreases (58, 59). Additionally, warm-up activates the nervous system, accelerating the central recruitment of muscles (59, 60).

### Interpretation of subgroup analyses

All subgroup analyses were exploratory and prespecified. The data cannot generate definitive conclusions regarding group differences, and it merely serves as an exploration of potential trends. As the interplay among these outcomes was not formally tested, our inferences remain hypothetical.

Preventive training program has less impact on the incidence of injuries in the formal competition compared to regular training (61). This may be attributed to the fact that exercise intensity during regular training is more readily controllable for athletes. In contrast, inappropriate or abruptly altered training loads are key contributors to athletic injuries (1). Indeed, any intensity of exercise necessitates a structured training process to facilitate physiological adaptation, which is a critical principle for mitigating injury risk during high stress periods (1). Prior research has demonstrated that athletes' injury risk is markedly higher in the first 3 weeks of a competitive season than in the subsequent 3-week period (62, 63). In actual competitive matches, athletes are more prone to sustaining injuries caused by physical contact (63). Injury prevention training can help athletes prepare for high-intensity athletic activities. By enhancing athletes' overall physical fitness and motor control, they can better manage unexpected injury risks. However, athletes often strive to achieve peak performance during competitions, occasionally exceeding the intensity thresholds established during routine training (64). As the level of competition that athletes participate in increases, the risk of getting injury increases (1, 64). Most injury prevention programs focus on regular training, so their effect during competitions remains limited.

Male and female athletes exhibit substantial differences in anthropometric and physiological characteristics. For instance, male athletes possess greater muscle mass and generate higher levels of muscular power, largely attributable to the effects of androgenic hormones (65, 66). On the other hand, female athletes tend to have relatively lower bone mineral density and joint stability, which increases their overall injury susceptibility (67–69). Preventive training helps female athletes reduce their risk of injury by enhancing strength, stability, and movement efficiency (70). Additionally, empirical research indicates that female athletes demonstrated greater injury risk awareness and exhibit a more risk-averse behavioral tendency during athletic participation (21). Another study reported that the preventive programs are better suited for female athletes (71). Female athletes may be more compliant with training guidelines.

Furthermore, basketball players appeared to derive greater benefits from injury prevention training compared with athletes from other sports disciplines. The findings for basketball players are more robust, owing to the larger sample size in this subgroup (a 2:5 ratio relative to soccer players). In contrast, the intervention effects were not statistically significant among soccer players, suggesting that additional research is warranted to optimize injury prevention programs for athletes whose performance relies heavily on lower-extremity function.

### Heterogeneity and methodological considerations

High heterogeneity persisted across the included studies and could not be explained by subgroup exploration. Therefore, we could only propose several hypothetical explanations for this heterogeneity: differences among studies in participants' geography, age, sex distribution, competitive level and sport discipline. Meanwhile, discrepancies in the definitions of overall injury across different studies may also give rise to heterogeneity. This heterogeneity might limit the interpretability of our pooled estimates.

### Practical implications

Knee injuries are common and often career-limiting. This study systematically reviewed 10 RCTs that evaluated the effect of lower-limb injury-prevention programs in athletes. The findings help to create better injuries prevention plans in the future. In addition, injury prevention training does not require special equipment or facilities, making it easy for clubs or teams to implement at a low cost. For athletes, using a more scientific training programs can be very helpful for their careers. For coaches and fitness practitioners, this study offers actionable insights for the design of targeted training programs.

### Strengths and limitations

This meta-analysis designed to explore the effects of preventive training on knee joint injuries in athletes. Guided by the principles of evidence-based medicine, this study ensures that the conclusions are presented in a more clear and intuitive manner. Different from previous research, this study places greater emphasis on the impact of preventive training on the knee joint, and the analytical outcomes encompass all reported cases related to knee joint injuries. Meanwhile, to clarify the efficacy of training programs, this study intentionally conducted subgroup analyses stratifies by gender, injury occasion, and sport type. Although the number of original studies included may impose certain limitations on the reliability of the results, the findings still merit further in-depth research and deliberation.

However, this study has several limitations. First, substantial heterogeneity was observed across the included studies, attributable to variations in exercise modality, intensity, and intervention protocols. We attempted to explore the sources of such heterogeneity via subgroup analyses; nonetheless, the underlying causes remained elusive. Second, discrepancies existed in the study design of the included literature: nine studies were cluster RCTs, and one was an individual RCT. Although no outcomes were excluded during the sensitivity analysis, this methodological heterogeneity may have exerted a potential impact on the pooled results of the present study. And also, the power of publication bias tests was limited with small study numbers. Third, the definition of overall injury rate was not consistent across the included studies, and the analysis was performed using the number of injuries. Despite no articles being excluded in the sensitivity analysis, these findings should be interpreted with caution. Additionally, only injury counts were used in the analysis with no consideration for exposure time; this is an additional methodological limitation, and the results should thus be viewed cautiously. Fourth, the ambiguity of compliance and implementation may affect the effect size observed in our study. Meanwhile, constraints related to the databases and the language of the included articles may also compromise the richness of our research findings. To address limitations related to heterogeneity, subgroup classification, and variations in injury definitions, future research should prioritize additional randomized controlled trials or adopt network meta-analysis approaches. At the same time, more reasonable statistical methods for processing the data from clustering experiments were developed. These strategies may help to further validate and refine the conclusions drawn from the present study.

## Conclusion

Exercise-based injury-prevention programs that incorporate strength, balance and plyometric training can reduce the incidence of knee injuries, but the evidence is characterized by high statistical heterogeneity, variable effects across athlete subgroups, and differences in intervention content and study design. Consequently, any clinical benefit should be viewed as provisional, and well-designed trials are still needed to establish the precise frequency, intensity, and duration required for consistent protection.
