# Supplementary material for: Evaluating the effectiveness of preventive training programs in reducing the incidence of knee injuries: a systematic review and meta-analysis
Source: Front Public Health. 2026 Mar 23;14:1746109. doi: 10.3389/fpubh.2026.1746109 (PMC13050942; doi:10.3389/fpubh.2026.1746109)
Supplement: Supplementary file 1 [file Data_Sheet_1.docx]

**Search strategy**

**Pubmed:**

(((("Athletes"[Mesh]) OR ((((((((Athletes[Title/Abstract]) OR (Athlete[Title/Abstract])) OR (Professional Athletes[Title/Abstract])) OR (Professional Athlete[Title/Abstract])) OR (Elite Athletes[Title/Abstract])) OR (Elite Athlete[Title/Abstract])) OR (College Athletes[Title/Abstract])) OR (College Athlete[Title/Abstract]))) AND (("Knee Injuries"[Mesh]) OR ((((((((((((((((((((((((((((((((((((((((((((Knee Injuries[Title/Abstract]) OR (Knee Injury[Title/Abstract])) OR (Knee Dislocation[Title/Abstract])) OR (Knee Dislocations[Title/Abstract])) OR (Patellar Dislocation[Title/Abstract])) OR (Patellar Dislocations[Title/Abstract])) OR (Osteoarthritis, Knee[Title/Abstract])) OR (Knee Osteoarthritis[Title/Abstract])) OR (Osteoarthritis of Knee[Title/Abstract])) OR (Osteoarthritis of the Knee[Title/Abstract])) OR (Knee Fractures[Title/Abstract])) OR (Knee Fracture[Title/Abstract])) OR (Knee Joint Fractures[Title/Abstract])) OR (Joint Fracture, Knee[Title/Abstract])) OR (Knee Joint Fracture[Title/Abstract])) OR (Tibial Spine Fractures[Title/Abstract])) OR (Spine Fracture, Tibial[Title/Abstract])) OR (Tibial Spine Fracture[Title/Abstract])) OR (Tibial Eminence Fractures[Title/Abstract])) OR (Eminence Fracture, Tibial[Title/Abstract])) OR (Tibial Eminence Fracture[Title/Abstract])) OR (Tibial Tuberosity Fractures[Title/Abstract])) OR (Tibial Tuberosity Fracture[Title/Abstract])) OR (Fractured Knee[Title/Abstract])) OR (Fractured Knees[Title/Abstract])) OR (Femoral Condyle Fractures[Title/Abstract])) OR (Femoral Condyle Fracture[Title/Abstract])) OR (Femoral Condyles Fractures[Title/Abstract])) OR (Femoral Condyles Fracture[Title/Abstract])) OR (Fracture, Femoral Condyles[Title/Abstract])) OR (Patellofemoral Pain Syndrome[Title/Abstract])) OR (Anterior Knee Pain Syndrome[Title/Abstract])) OR (Patellofemoral Syndrome[Title/Abstract])) OR (Patellofemoral Pain[Title/Abstract])) OR (Patellofemoral Pains[Title/Abstract])) OR (Anterior Cruciate Ligament Injuries[Title/Abstract])) OR (ACL Injuries[Title/Abstract])) OR (ACL Injury[Title/Abstract])) OR (Anterior Cruciate Ligament Injury[Title/Abstract])) OR (Anterior Cruciate Ligament Tear[Title/Abstract])) OR (ACL Tears[Title/Abstract])) OR (ACL Tear[Title/Abstract])) OR (Anterior Cruciate Ligament Tears[Title/Abstract])) OR (Iliotibial Band Syndrome[Title/Abstract])))) AND (("prevention and control" [Subheading]) OR ((((((prevention[Title/Abstract] AND control[Title/Abstract]) OR (prophylaxis[Title/Abstract])) OR (preventive therapy[Title/Abstract])) OR (preventive measures[Title/Abstract])) OR (prevention[Title/Abstract])) OR (control[Title/Abstract])))) AND ((((((((((((Random Allocation[Title/Abstract]) OR (placebos[Title/Abstract])) OR (Clinical Trials, Randomized[Title/Abstract])) OR (Trials, Randomized Clinical[Title/Abstract])) OR (Controlled Clinical Trials, Randomized[Title/Abstract])) OR (Allocation, Random[Title/Abstract])) OR (Randomization[Title/Abstract])) OR (randomized[Title/Abstract])) OR (randomised[Title/Abstract])) OR (randomly[Title/Abstract])) OR (trial[Title/Abstract])) OR (phase[Title/Abstract]))

**WOS:**

prevention and control (Topic) or prophylaxis (Topic) or preventive therapy (Topic) or preventive measures (Topic) or prevention (Topic) or control (Topic) And Random Allocation (Topic) or placebos (Topic) or Clinical Trials, Randomized (Topic) or Trials, Randomized Clinical (Topic) or Controlled Clinical Trials, Randomized (Topic) or Allocation, Random (Topic) or Randomization (Topic) or randomized (Topic) or randomised (Topic) or randomly (Topic) or trial (Topic) or phase (Topic) And Knee Injuries (Topic) or Knee Injury (Topic) or Knee Dislocation (Topic) or Knee Dislocations (Topic) or Patellar Dislocation (Topic) or Patellar Dislocations (Topic) or Osteoarthritis, Knee (Topic) or Knee Osteoarthritis (Topic) or Osteoarthritis of Knee (Topic) or Osteoarthritis of the Knee (Topic) or Knee Fractures (Topic) or Knee Fracture (Topic) or Knee Joint Fractures (Topic) or Joint Fracture, Knee (Topic) or Knee Joint Fracture (Topic) or Tibial Spine Fractures (Topic) or Spine Fracture, Tibial (Topic) or Tibial Spine Fracture (Topic) or Tibial Eminence Fractures (Topic) or Eminence Fracture, Tibial (Topic) or Tibial Eminence Fracture (Topic) or Tibial Tuberosity Fractures (Topic) or Tibial Tuberosity Fracture (Topic) or Fractured Knee (Topic) or Fractured Knees (Topic) or Femoral Condyle Fractures (Topic) or Femoral Condyle Fracture (Topic) or Femoral Condyles Fractures (Topic) or Femoral Condyles Fracture (Topic) or Fracture, Femoral Condyles (Topic) or Patellofemoral Pain Syndrome (Topic) or Anterior Knee Pain Syndrome (Topic) or Patellofemoral Syndrome (Topic) or Patellofemoral Pain (Topic) or Patellofemoral Pains (Topic) or Anterior Cruciate Ligament Injuries (Topic) or ACL Injuries (Topic) or ACL Injury (Topic) or Anterior Cruciate Ligament Injury (Topic) or Anterior Cruciate Ligament Tear (Topic) or ACL Tears (Topic) or ACL Tear (Topic) or Anterior Cruciate Ligament Tears (Topic) or Iliotibial Band Syndrome (Topic) And Athletes (Topic) or Athlete (Topic) or Professional Athletes (Topic) or Professional Athlete (Topic) or Elite Athletes (Topic) or Elite Athlete (Topic) or College Athletes (Topic) or College Athlete (Topic)

**Embase:**

'athlete'/exp or athlete:ti,ab,kw OR athletes:ti,ab,kw OR sportman:ti,ab,kw OR sportmen:ti,ab,kw OR 'sports player':ti,ab,kw OR 'sports players':ti,ab,kw OR sportsman:ti,ab,kw OR sportsmen:ti,ab,kw OR sportspeople:ti,ab,kw OR sportsperson:ti,ab,kw OR sportspersons:ti,ab,kw OR sportsplayers:ti,ab,kw OR sportswoman:ti,ab,kw OR sportswomen:ti,ab,kw OR sportwomen:ti,ab,kw

And

'random allocation':ti,ab,kw OR placebos:ti,ab,kw OR 'clinical trials, randomized':ti,ab,kw OR 'trials, randomized clinical':ti,ab,kw OR 'controlled clinical trials, randomized':ti,ab,kw OR 'allocation, random':ti,ab,kw OR randomization:ti,ab,kw OR randomized:ti,ab,kw OR randomised:ti,ab,kw OR randomly:ti,ab,kw OR trial:ti,ab,kw OR phase:ti,ab,kw

And

'knee injury':ti,ab,kw OR 'knee injuries':ti,ab,kw OR 'knee joint injury':ti,ab,kw OR 'knee joint trauma':ti,ab,kw OR 'knee open injury':ti,ab,kw OR 'knee trauma':ti,ab,kw OR 'knee dislocation':ti,ab,kw OR 'knee joint dislocation':ti,ab,kw OR 'patella dislocation':ti,ab,kw OR 'patella luxation':ti,ab,kw OR 'patella recurrent dislocation':ti,ab,kw OR 'patella subluxation':ti,ab,kw OR 'patellar dislocation':ti,ab,kw OR 'knee osteoarthritis':ti,ab,kw OR 'femorotibial arthrosis':ti,ab,kw OR gonarthrosis:ti,ab,kw OR 'knee arthrosis':ti,ab,kw OR 'knee joint arthrosis':ti,ab,kw OR 'knee joint osteoarthritis':ti,ab,kw OR 'knee osteo-arthritis':ti,ab,kw OR 'knee osteo-arthrosis':ti,ab,kw OR 'knee osteoarthrosis':ti,ab,kw OR 'knee fracture':ti,ab,kw OR 'broken knee':ti,ab,kw OR 'fractured knee':ti,ab,kw OR 'knee fractures':ti,ab,kw OR 'knee joint fracture':ti,ab,kw OR 'patellofemoral pain syndrome':ti,ab,kw OR 'anterior cruciate ligament injury':ti,ab,kw OR 'anterior cruciate ligament injuries':ti,ab,kw OR 'iliotibial band friction syndrome':ti,ab,kw OR 'iliotibial band syndrome':ti,ab,kw or 'knee injury'/exp

And

prophylaxis:ti,ab,kw OR 'disease prevention':ti,ab,kw OR 'disease prophylaxis':ti,ab,kw OR 'health protection':ti,ab,kw OR 'preventive medication':ti,ab,kw OR 'preventive therapy':ti,ab,kw OR 'preventive treatment':ti,ab,kw OR 'prophylactic institution':ti,ab,kw OR 'prophylactic management':ti,ab,kw OR 'prophylactic medication':ti,ab,kw OR 'prophylactic therapy':ti,ab,kw OR 'prophylactic treatment':ti,ab,kw OR prevention:ti,ab,kw OR (prevention:ti,ab,kw AND control:ti,ab,kw) or 'prophylaxis'/exp

**Cochrane:**

Athletes or (Athletes):ti,ab,kw OR (Athlete):ti,ab,kw OR (Professional Athletes):ti,ab,kw OR (Professional Athlete):ti,ab,kw OR (Elite Athletes):ti,ab,kw or (Elite Athlete):ti,ab,kw OR (College Athletes):ti,ab,kw OR (College Athlete):ti,ab,kw

And

Knee Injuries or (Knee Injuries):ti,ab,kw OR (Knee Injury):ti,ab,kw OR (Knee Dislocation):ti,ab,kw OR (Knee Dislocations):ti,ab,kw OR (Patellar Dislocation):ti,ab,kw or (Patellar Dislocations):ti,ab,kw OR (Osteoarthritis, Knee):ti,ab,kw OR (Knee Osteoarthritis):ti,ab,kw OR (Osteoarthritis of Knee):ti,ab,kw OR (Osteoarthritis of the Knee):ti,ab,kw or (Knee Fractures):ti,ab,kw OR (Knee Fracture):ti,ab,kw OR (Knee Joint Fractures):ti,ab,kw OR (Joint Fracture, Knee):ti,ab,kw OR (Knee Joint Fracture):ti,ab,kw or (Tibial Spine Fractures):ti,ab,kw OR (Spine Fracture, Tibial):ti,ab,kw OR (Tibial Spine Fracture):ti,ab,kw OR (Tibial Eminence Fractures):ti,ab,kw OR (Eminence Fracture, Tibial):ti,ab,kw or (Tibial Eminence Fracture):ti,ab,kw OR (Tibial Tuberosity Fractures):ti,ab,kw OR (Tibial Tuberosity Fracture):ti,ab,kw OR (Fractured Knee):ti,ab,kw OR (Fractured Knees):ti,ab,kw or (Femoral Condyle Fractures):ti,ab,kw OR (Femoral Condyle Fracture):ti,ab,kw OR (Femoral Condyles Fractures):ti,ab,kw OR (Femoral Condyles Fracture):ti,ab,kw OR (Fracture, Femoral Condyles):ti,ab,kw or (Patellofemoral Pain Syndrome):ti,ab,kw OR (Anterior Knee Pain Syndrome):ti,ab,kw OR (Patellofemoral Syndrome):ti,ab,kw OR (Patellofemoral Pain):ti,ab,kw OR (Patellofemoral Pains):ti,ab,kw or (Anterior Cruciate Ligament Injuries):ti,ab,kw OR (ACL Injuries):ti,ab,kw OR (ACL Injury):ti,ab,kw OR (Anterior Cruciate Ligament Injury):ti,ab,kw OR (Anterior Cruciate Ligament Tear):ti,ab,kw or (ACL Tears):ti,ab,kw OR (ACL Tear):ti,ab,kw OR (Anterior Cruciate Ligament Tears):ti,ab,kw OR (Iliotibial Band Syndrome):ti,ab,kw

And

prevention & control or (prevention and control):ti,ab,kw OR (prophylaxis):ti,ab,kw OR (preventive therapy):ti,ab,kw OR (preventive measures):ti,ab,kw OR (prevention):ti,ab,kw or (control):ti,ab,kw

And

(Random Allocation):ti,ab,kw OR (placebos):ti,ab,kw OR (Clinical Trials, Randomized):ti,ab,kw OR (Trials, Randomized Clinical):ti,ab,kw OR (Controlled Clinical Trials, Randomized):ti,ab,kw or (Allocation, Random):ti,ab,kw OR (Randomization):ti,ab,kw OR (randomized):ti,ab,kw OR (randomised):ti,ab,kw OR (randomly):ti,ab,kw or (trial):ti,ab,kw OR (phase):ti,ab,kw
