# Supplementary material for: Evaluating the effectiveness of preventive training programs in reducing the incidence of knee injuries: a systematic review and meta-analysis
Source: Front Public Health. 2026 Mar 23;14:1746109. doi: 10.3389/fpubh.2026.1746109 (PMC13050942; doi:10.3389/fpubh.2026.1746109)

Sensitivity analysis

knee injury risk

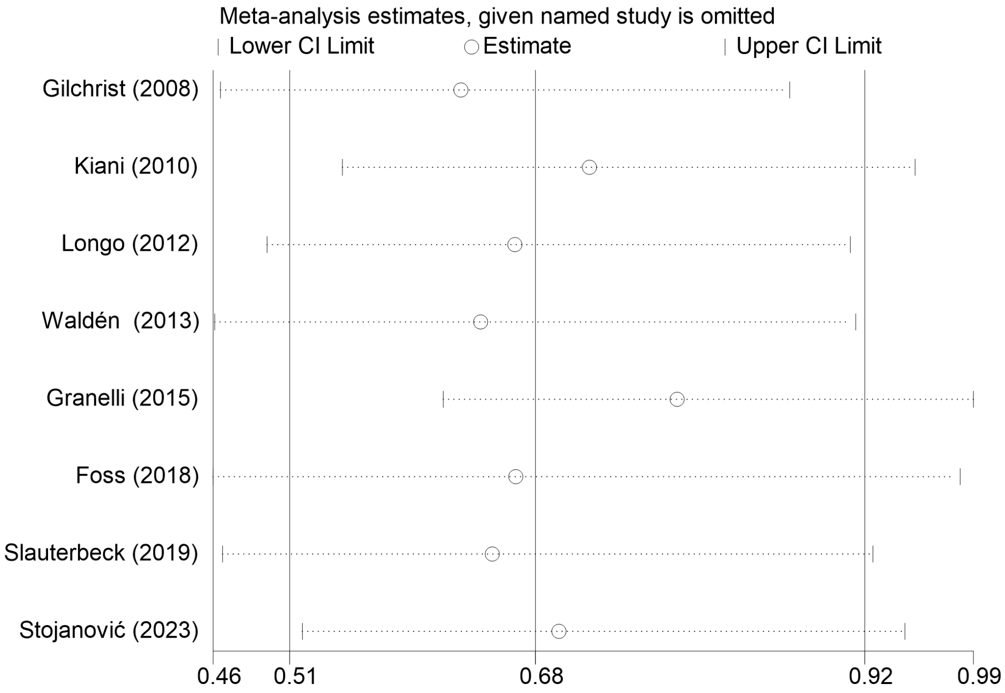

Anterior Cruciate Ligament injury risk

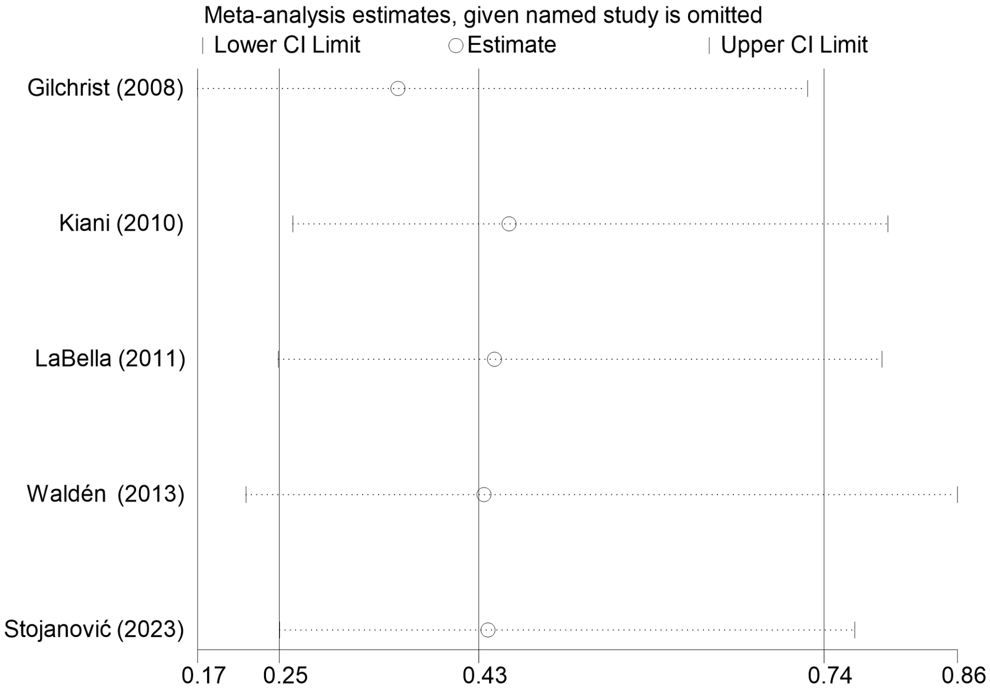

injury risk stratified by time of injury (practice vs. game)

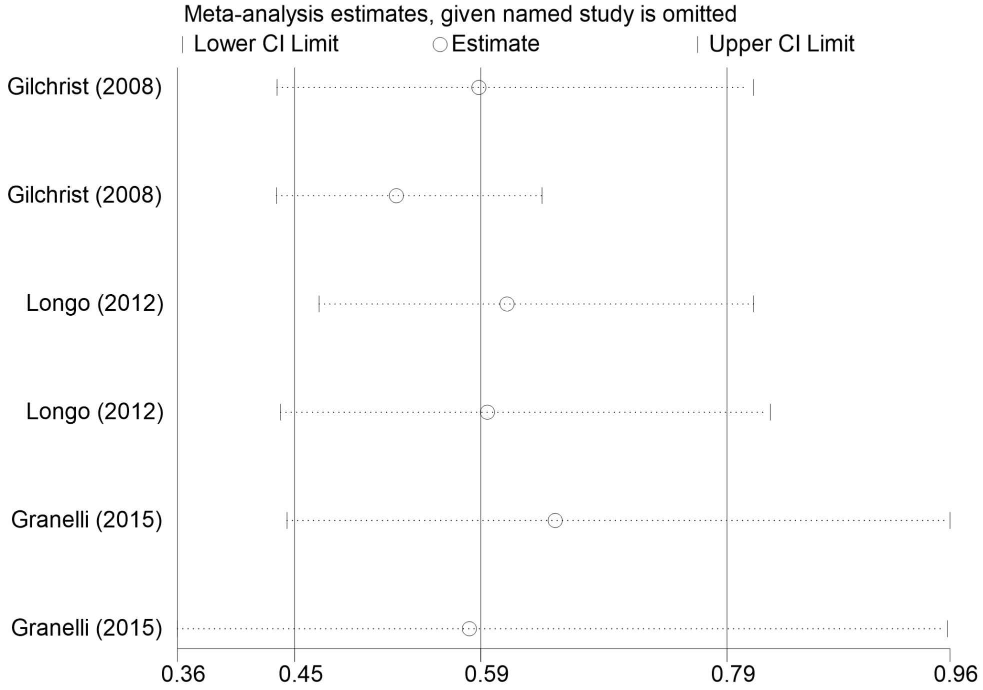

injury risk Stratified by type of athlete

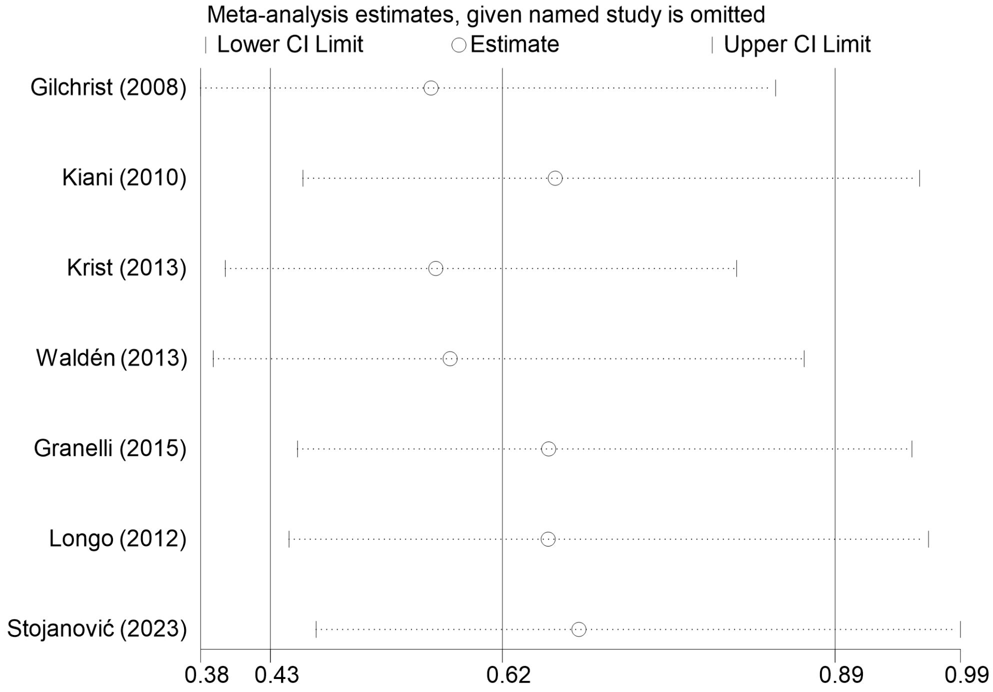

injury risk Stratified by sex

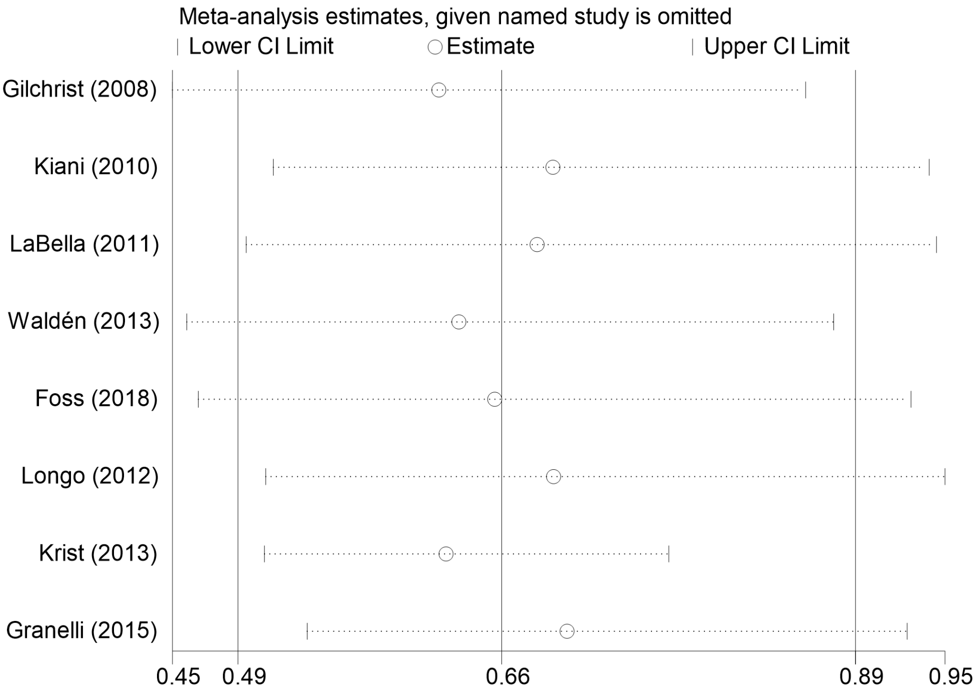

Supplement: Supplementary file 2 [file Data_Sheet_2.pdf]
